# Ambulatory Blood Pressure Monitoring: Five Decades of More Light and Less Shadows

**DOI:** 10.5935/abc.20160065

**Published:** 2016-06

**Authors:** Fernando Nobre, Décio Mion Junior

**Affiliations:** 1 Faculdade de Medicina de Ribeirão Preto da Universidade de São Paulo - Ribeirão Preto, SP - Brazil; 2 Faculdade de Medicina da Universidade de São Paulo, SP - Brazil

**Keywords:** Blood Pressure Monitoring, Ambulatory / trends, Hypertension, White Coat Hypertension, Medication Therapy Management

## Abstract

Casual blood pressure measurements have been extensively questioned over the last five decades. A significant percentage of patients have different blood pressure readings when examined in the office or outside it. For this reason, a change in the paradigm of the best manner to assess blood pressure has been observed. The method that has been most widely used is the Ambulatory Blood Pressure Monitoring - ABPM. The method allows recording blood pressure measures in 24 hours and evaluating various parameters such as mean BP, pressure loads, areas under the curve, variations between daytime and nighttime, pulse pressure variability etc. Blood pressure measurements obtained by ABPM are better correlated, for example, with the risks of hypertension. The main indications for ABPM are: suspected white coat hypertension and masked hypertension, evaluation of the efficacy of the antihypertensive therapy in 24 hours, and evaluation of symptoms. There is increasing evidence that the use of ABPM has contributed to the assessment of blood pressure behaviors, establishment of diagnoses, prognosis and the efficacy of antihypertensive therapy. There is no doubt that the study of 24-hour blood pressure behavior and its variations by ABPM has brought more light and less darkness to the field, which justifies the title of this review.

"Indeed, it is somewhat paradoxical that a clinical condition such as arterial hypertension, which is defined in terms of blood pressure values only, may be diagnosed on the basis of few occasional blood pressure measurements, and that life-long treatment is often instituted following measurements taken over just a few minutes"

Alberto Zanchetti

(AJH 1997; 10:1068-1080)

## Introduction

Since Riva-Roc ci^1^ created the sphygmomanometer in 1886, casual blood pressure measurement have been used for the assessment of blood pressure and establishment of diagnosis, prognosis, efficacy and treatment of hypertension. However, the value of casual blood pressure has been questioned in all these contexts in the last five decades.

Since the study published by Ai man & Goldshi ne in 1940,^2^ it has been known that a significant percentage of patients have higher blood pressure measures when they are taken in the clinic setting than at home. In addition, blood pressure measures taken by different observers - the patient, the physician, or the nurse - are also different, particularly when taken by the physician, who obtains the highest measures.^3,4^ This may lead to erroneous blood pressure readings, incorrect diagnosis and inappropriate management of the disease.^5,6^

These aspects have changed the paradigm of the best method to assess blood pressure behavior. The ambulatory blood pressure monitoring - ABPM is the method of choice for 24 hour-blood pressure monitoring considering its advantages established in previous reviews and guidelines.^7-11^

This is especially due to advances in the techniques for 24-blood pressure monitoring and use of state-of-the-art equipment, which have been more appropriate, easier to use, relatively low cost, validated by strict international protocols, automatic, and electronically sophisticated, offering reliable performance.^12^

Another reason for the increasing use of ABPM is the evidence that blood pressure readings obtained by this method are more correlated with the effects of hypertension, as compared with others.^13-15^

### The history of ABPM

In the 60's decade (i.e. five decades ago), Kain et al.^16^ demonstrated the benefits of ABPM, and the attractive possibility of measuring blood pressure during patients' daily activities.

According to a search performed on MEDLINE database on May 11, 2015, since 2001, more than 2000 articles have been published every five years, showing the importance of this revolutionary method in the establishment of diagnosis and prognosis of patients with altered blood pressure, and in the assessment of the antihypertensive therapy. The first study, published in 1962, was crucial for demonstrating the assessment of 24-hour blood pressure without an observer, using a semi-automatic method.^17^

**Figure 1:**
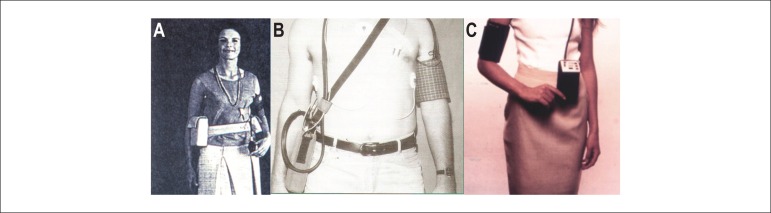
From left to right: 24-hour blood pressure monitoring devices used in 1966 (A), 1988 (B) and 2015 (C) (Authors' personal archive).

Figure 1 depicts a sequence of 24-hour blood pressure monitors in three different moments, and the evolution of these devices over time.

**Figure 2:**
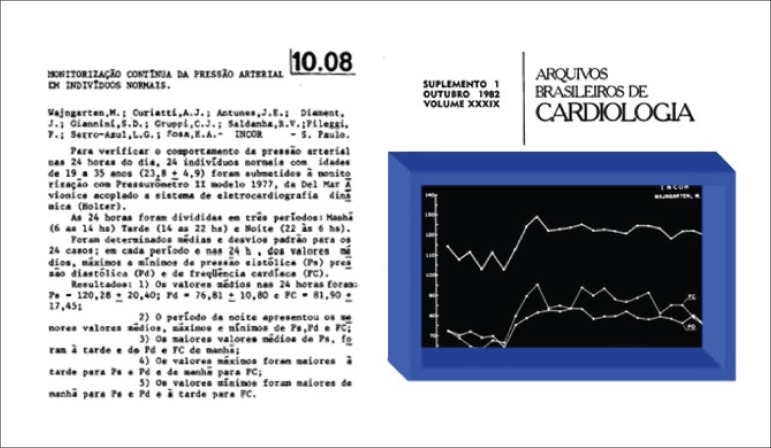
Continuous blood pressure monitoring in healthy subjects (presented in the in the Brazilian Congress of Cardiology in 1982).

The use of ABPM has been consolidated in Brazil, similarly to what has occurred in the world. In 1982, Prof. Mauricio Wajngarten and colleagues presented, for the first time, a 24-hour blood pressure recording in the Brazilian Congress of Cardiology (Figure 2).

The use of ABPM has spread in our community by means of courses offered throughout the country. One example was the PRONAM - *Programa Nacional de Atualização em MAPA e Hipertensão* (National Program for ABPM and Hypertension Update), an on-site course, run by the authors in more than 150 editions from 1996. The program has been run by the Corporate University of the Brazilian Society of Cardiology since 2011, as one of the strategies of distance education in cardiology.

Besides, under our supervision and with the contribution of specialists in the field, five editions of the book *MAPA* - *Monitorização Ambulatorial da Pressão Arterial* (ABPM - Ambulatory Blood Pressure Monitoring) were published in 1995, 1998, 2004, 2007 and 2014. One of them was translated to Spanish and offered in Spanish-speaking countries in 2001.

### ABPM in our days

The Brazilian Societies of Cardiology, Hypertension and Nephrology have published guidelines on ABPM since 1993.^18-22^

Additionally, international guidelines that regulated the (rational and scientifically correct) use of ABPM,^8-10,23-25^ including in children and adolescents^26^, have contributed to a broad, consistent use of the method.

Nowadays, it is possible to monitor blood pressure measures during 24-hour periods or longer, with assessment of hemodynamic parameters that reflect blood pressure fluctuations: mean systolic and diastolic blood pressures, pressure overload, areas under the curve, blood pressure changes between sleep and wakefulness, blood pressure variability, pulse pressure, among others. These data may be represented in an analytical summary or graphics showing the variability of blood pressure by time.^27^

Therefore, the use of ABPM has considerably increased. This is explained by the fact that the measures obtained by ABPM better reflect blood pressure behavior. Also, the development of more comfortable, reliable, safer devices significantly decreased the limitations for the routine use of the method.

 The increasing use of ABPM in clinical practice may increase, since health insurance plans from all over the world, probably motivated by these data, have added ABPM to the list of exams considered as 'useful' and acceptable to be performed.

### Indications, advantages and limitations

The indications, advantages and limitations of ABPM, according to the V Brazilian guidelines for the use of ABPM^22^ are described in Tables I, II and III.

**Table I:** Main indications for 24-hour ambulatory blood pressure monitoring^22^

1. Suspected white coat hypertension (Recommendation grade I, level of evidence A)
2. Assessment of normotensive patients with target-organ lesions at the physician's office, i.e. wit suspected masked hypertension (Recommendationgrade I, level of evidence A)
3. Evaluation of the efficacy of the antihypertensive therapy: a) When casual blood pressure remains elevated despite optimization of the antihypertensive therapy for the diagnosis of persistent hypertension (Recommendation grade IIa, level of evidence B) or white coat effect (Recommendation grade IIa, level of evidence B), or b) When casual blood pressure is controlled and there are signs of persistence (Recommendation grade IIa, level of evidence B), or progression(Recommendation grade I, level of evidence B) of target-organ lesions
1. Evaluation of symptoms, specially hypotension (Recommendation grade I, level of evidence D)

**Table II:** Main advantages of 24-hour ambulatory blood pressure monitoring^22^

1. Multiple measures of blood pressure for 24 hours. Assessment of blood pressure during daily activities and during sleep.
2. Assessment of blood pressure circadian rhythm
3. Assessment of blood pressure means, overload and variability. Identificationof "alarming reaction"
4. Placebo effect reduction
5. Assessment of the antihypertensive effect in 24 hours
6. Possibility of risk stratification

**Table III:** Limitations of 24-hour ambulatory blood pressure monitoring^22^ (Recommendation grade I, level of evidence D)

1. When the cuff cannot be adjusted due to arm circumference
2. When systolic pressure values are very high
3. Clinical situations associated with movement disorders (e.g. Parkinson's disease)
4. When pulse is irregular due to cardiac arrhythmias (atrial fibrillation and atrial flutter)
5. Presence of auscultatory gaps during manual measurement of blood pressure

With respect to the indication of ABPM, it is worth mentioning that in 2001, i.e., more than one decade ago, the Centers for Medicare and Medcaid Services recommended the reimbursement of the exam cost for patients with suspected white coat hypertension.^28^ In 2011, the National Institute for Health and Care Excellence (NICE) recommended the use of ABPM for all individuals with blood pressure ≥ 140/ 90 mm Hg, measured at the physician's office, for considering it a cost-effective procedure^29^. This recommendation allows the diagnosis of white coat hypertension, with cost savings, according to a study that used a cost-effectiveness analysis, based on the probabilistic Markov model.^30^ However, patients with marked hypertension are not included in NICE recommendation, since they are normotensive in the physician's office. This situation tends to be solved as the costs of the ABPM decreases, and the exam may be indicated for hypertensive and normotensive patients.^31^

### ABPM and its contribution for the assessment of blood pressure behavior and establishment of diagnosis

The use of ABPM in the assessment of blood pressure behaviors has spread and been corroborated by national^18-22^ and international^8-10,23-27^ guidelines. In general, the main objective of using ABPM is based on the decision whether or not to treat the patient on the basis of his/her blood pressure measures. Considering that the beginning of the antihypertensive therapy will be based on blood pressure measures, two types of error, undesirable and potentially harmful to the patient may occur in case the values do not represent the real behavior of blood pressure. First, if casual blood pressure, i.e. taken in the physician's office, overestimates the real value, therapy may be unnecessarily started; on the other hand, if it underestimates the real value, the patient may be deprived of a beneficial therapy. Therefore, it is crucial to obtain reliable values, truly representative of blood pressure behavior.

Thanks to the use of ABPM, today we know that blood pressure values obtained in the office setting may be higher, similar or lower than those obtained by the method. From these differences, four diagnosis may be identified: normotension, hypertension, white coat hypertension (detected in the physician's office only), and masked hypertension (white coat normotension).^22^

Normotension is characterized by normal blood pressure values in the office (< 140/90 mmHg) and in 24-hour ABPM (≤ 125/75 mmHg), while hypertension is characterized by abnormal blood pressure values in the office (≥ 140/90 mmHg) and in ABPM (≥ 130/85 mmHg).^22^

**Figure 3:**
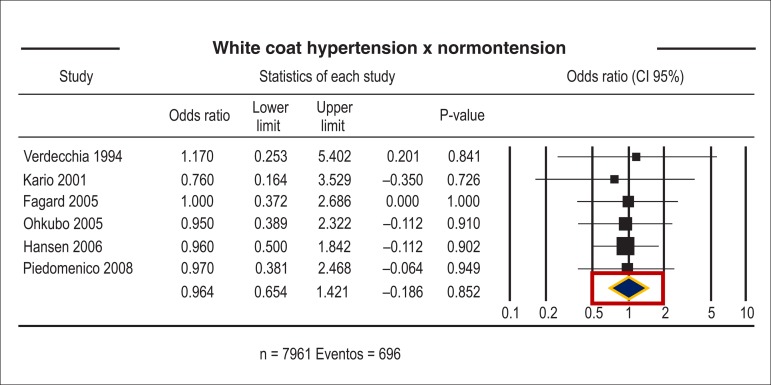
Odds ratio of patients with white coat hypertension compared with normotensive patients.^34^

White coat hypertension occurs in 15-30% of individuals with elevated blood pressure in the office setting.^8^ It occurs when abnormal blood pressure values are obtained in the office (≥ 140/90 mm Hg) and normal values are obtained during the ABPM (≤ 135/85 mm Hg).^22,32^ Interestingly, in this case, there is a change from the diagnosis of normotension detected out of the office setting to the diagnosis of hypertension detected in the office. Since there are no pathognomonic signs of white coat hypertension, the most common characteristics that help in the diagnosis are: elderly patients, women, pregnant women, non-smokers, patients with diagnosis of stage 1 hypertension after blood pressure readings in the office, and individuals without target-organ lesions.^33^ The attributable risk of white coat hypertension has been extensively discussed.^32^ Some studies have indicated that white coat hypertension has an intermediate cardiovascular risk, between normotension and hypertension, closer to normotension though (Figure 3).^34^ The IDACO study, a cohort study involving 7,295 persons followed for 10.6 years, showed that the incidence of cardiovascular events in untreated subjects with white coat hypertension was not different from that observed in normotensive, untreated subjects.^35^ There is no evidence of benefit from interventions in this group of patients.^32^ These patients need to be followed, and the change of life habits is imperative.^8,32^ It is recommended that the diagnosis of white coat hypertension be confirmed within 3-6 months, and the patient should be followed every year by ABPM to detect progression of hypertension, since these patients have a higher probability to develop established hypertension.^8^

On the other hand, the white coat effect or white coat phenomenon is defined by the difference between office blood pressure and ambulatory (ABPM) blood pressure, without changing the diagnosis from normotension to hypertension. When the differences are higher than 20 and 10 mm Hg for systolic and diastolic pressure, respectively, the white coat effect is considered significant. It occurs in almost all individuals, at higher or lower degrees,^36^ with a mean of 27 mmHg increase in systolic blood pressure.^4,32^

**Figure 4:**
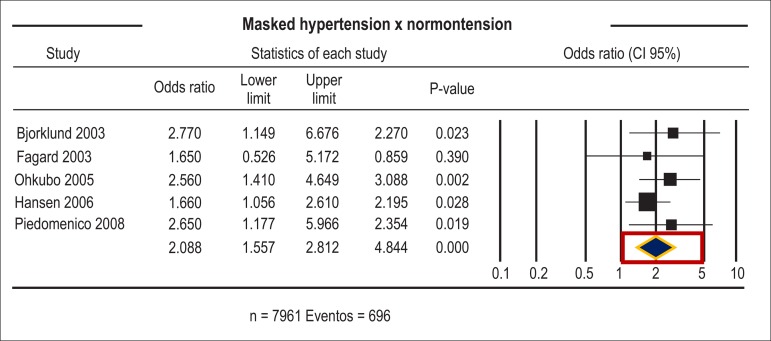
Odds ratio of patients with masked hypertension compared with normotensive patients.^34^

Masked hypertension of white coat normotension occurs in 10-40% of patients not receiving anti-hypertensive therapy.^37,38^ It is defined by the presence of normal blood pressure values obtained in the office (< 140/90 mmHg) and abnormal ABPM values (> 130/85 mm Hg).^22^ There is a change of diagnosis from hypertension during daily living to normotension in the office setting. Multivariate analysis studies have identified as associated risk factors: masked hypertension, male sex, smoking, and body mass index.^39^ Masked hypertension is associated with increased risk of cardiovascular morbidity and mortality. However, since office measures are normal, this risk may be underestimated.^40^ A meta-analysis of 12 studies, involving 4,884 untreated subjects - 2,467 normotensive, 1,641 hypertensive subjects, and 776 with masked hypertension - showed an association between masked hypertension and increased risk of structural changes in left ventricle. The risk observed in subjects with masked hypertension is nearly twice as high as that among normotensive subjects (Figure 4).^34^ The anti-hypertensive therapy seems to be the rational choice for these patients, although no randomized studies evaluating this procedure have been performed so far.^37,38^

### ABPM and prognosis of patient with arterial hypertension

Perloff et al,^42^ in 1983, were pioneers in assessing more than one thousand hypertensive patients by ABPM and by office measurements, and showed that ABPM measures are an independent indicator of prognosis. Twenty-four hour-values were more consistent than casual or office blood pressure in determining the risk level.

Longitudinal studies have given irrefutable evidence of the independent association between ABPM blood pressure and the risk for cardiovascular disease in the general population and in hypertensive individuals.^13-15^ Based on these studies, the ABPM has been considered a more consistent risk marker as compared with conventional methods to measure blood pressure.

Some parameters obtained by 24-hour ABPM may contribute to evaluate the prognosis. They will be individually evaluated, as follows:

### Mean arterial pressure

Cardiovascular risk is better correlated with 24-hour mean arterial pressure values than with office blood pressure.^43-46^ Conen & Bamberg^47^ showed, in a meta-analysis, that a 10-mmHg increase in 24-hour systolic pressure is associated with a 27% elevation of the risk for cardiovascular events, regardless of office blood pressure. In another meta-analysis, Fagard et al.^15^ analyzed four prospective studies conducted in Europe, and showed that daytime and nighttime blood pressure measured by 24-hour ABPM have a prognostic value for cardiovascular mortality, coronary disease, and stroke, independently of office blood pressure. Nighttime pressure and the night- day blood pressure ratio showed a prognostic value for all outcomes, whereas daytime blood pressure did not add prognostic precision to nighttime pressure. This corroborates the importance of ABPM, since this is the only non-invasive method to measure ambulatory blood pressure during sleep time.

Taken together, these evidences suggest that blood pressure values obtained by ABPM provide a better correlation with causal measures for total, cardiac and cerebrovascular risk.^46^

### Relationship between sleep and wakefulness

ABPM is the only method to assess arterial pressure during sleep and the blood pressure behavior between daytime and nighttime in a 24-hour period.

O'Brien et al,^48^ in 1988, in a letter published in The Lancet, suggested that patients who do not demonstrate a drop of 10% or more in blood pressure values between daytime and nighttime have a higher risk for cerebrovascular accident.

The decrease in blood pressure during sleep can be calculated by (mean daytime pressure - mean nighttime pressure) x 100 ÷ mean daytime pressure. Thus, according to this calculation of pressure reduction between daytime and nighttime, individuals may be classified as: dippers (≥ 10%), nondippers (< 10%), reverse dippers (≤ 0%) or extreme dippers (≥ 20%).^22^

There are evidences that 24-hour blood pressure behavior, considering these both periods of the day, is important for the prognosis.^49^ Ben-Dov et al.^50^ followed 3957 for a mean of 6.5 years, and observed that the mortality rate was higher in nondippers compared with dippers. Extreme dippers and dippers had similar risk. In another study,^51^ nondippers and reverse dippers had higher mortality risk. However, these individuals were older, and had a higher prevalence of non-white subjects, smokers, diabetes, hypertension, coronary disease, congestive heart failure and renal failure. Therefore, although nondipping and reverse dipping pose a higher mortality risk, this is associated with other cardiovascular risk factors.

In an international database including 8,711 individuals from 10 popuIations, isolated nighttime hypertension, i.e., subjects with increased blood pressure during sleep and normal awake blood pressure, was associated with increased total mortality risk and cardiovascular events. The mechanisms of nighttime hypertension and its correlation with poor prognostic have not been elucidated. Increased sympathetic activity, reduced baroreceptor sensitivity or autonomic dysfunction, a decrease in sodium excretion during daytime, nocturnal sodium excretion,^53^ increased activity during the night, sleep apnea, insulin resistance, endothelial dysfunction, or all of these factors may be involved.

With respect to siesta, in the study by Gomes, Pierin and Mion,^54^ 407 underwent ABPM during siesta (118 ± 58 minutes). Siesta had an effect on cardiac structural parameters, and on systolic and diastolic pressure during daytime. Patients with a 0-5% reduction in arterial pressure had thicker interventricular septum and posterior wall as compared with those with a reduction greater than 5%.

Then, the use of ABPM to assess the decrease in blood pressure and the mean values during sleep may provide important prognostic information for the clinical practice.

### Variability

The 24-hour ABPM offers an adequate short-term variability evaluation of between-measurement intervals not longer than 15 minutes. However, the method does not assess more complex parameters of blood pressure variability, including spectral index and analysis of baroreflex sensitivity, since it does not provide a beat-by-beat recording of arterial pressure.^8,55^

Longitudinal studies have shown that short-term variability may contribute to cardiovascular risk. Patients with increased arterial pressure variability have a higher risk for developing white coat hypertension of masked hypertension.^56,57^

More recently, a new index for short-term blood pressure variability has been proposed - the average real variability (ARV) - which is a more reliable representation of time series variability than standard deviation, and may be less sensitive to the relative low sampling frequency of the ABPM devices. The results suggest that the ARV adds prognostic value to the ABPM and may be used in therapeutic approaches to control blood pressure variability. It has been shown that 48 blood pressure readings in 24 hours were appropriate to calculate the ARV without loss or prognostic information.^58,59^

Blood pressure variability is not routinely assessed in ABPM, since its normal values have not been established. It is still unknown whether a reduction in short-term variability induced by the therapy is associated with a decrease in mortality and morbidity. Also, whether the antihypertensive therapy is indicated not only to reduce mean 24-hour blood pressure, but also to stabilize blood pressure and optimize cardiovascular protection. Dolan & O'Brien^60^ and Boggia et al.^61^ highlight that blood pressure variability on ABPM does not enhance the prediction of cardiovascular risk compared to the mean blood pressure, particularly in low-risk individuals.

### Pulse pressure

Pulse pressure has been considered an important prognostic marker, especially in patients aged greater than 55 years.^50^ It should be mentioned, however, that this measure is strongly influenced by an alerting reaction during measuring by the physician in the office, especially concerning systolic arterial pressure. Thus, measurements of pulse pressure in the office setting may be overestimated. Verdecchia et al.^63^ studied 2010 patients using ABPM and, according to the tertile distribution of pulse pressure distribution, the rate of total cardiovascular events was 1.19; 1.81 and 4.92, and that of fatal events was 0.11, 0.17 and 1.23. In these studies, patients with pulse pressure by ABPM higher than 53 mmHg were considered of high risk. Prospective, well-designed studies using ABPM are needed to establish the real prospective meaning of pulse pressure in the general population.

### Area under the pressure curve

Areas under the pressure curve have been studied by Nobre and Mion,^64^ who showed a direct relationship between the areas and left ventricle mass. Thus, these areas may be used as a parameter in the assessment of blood pressure behavior and target-organ lesions.

### ABPM and evaluation of the antihypertensive therapy efficacy

The need of an adequate control of blood pressure in 24 hours is well-established. The assessment and follow-up of hypertensive patients under treatment, using ABPM, seems to be more efficient than office measurements.^53^

Nonetheless, two issues need to be considered. First, will the cost of ABPM for hypertension control in treated patients be higher compared with office measurements? Second, is there any evidence that treated patients with controlled hypertension based on ABPM information will have a better prognosis, expressed by lower morbidity and mortality rates?

Regarding the first issue, Staes sen et al.^66^ showed, in an elegant study published in 1997, involving 419 hypertensive patients receiving antihypertensive drug treatment (213 based on ABPM compared with 206 based on office measurements), that the cost of the use of ABPM was not higher than office measurements during the study period. This was explained by the fact that individuals with white coat hypertension were excluded from the group receiving antihypertensive therapy, the number of antihypertensive drugs was lower in the group monitored by ABPM, and the number of physician visits was lower in the ABPM group as compared with the group monitored by office measurements. Cost analysis in both groups revealed that the costs of ABPM were outweighed by less intensive drug treatment and fewer physician visits.

With respect to the second issue, Schrader et al.^67^ demonstrated in a prospective, randomized study on 851 patients, that morbidity and mortality were lower in patients that underwent ABPM for the evaluation of antihypertensive treatment. A total of 1298 patients were included in the study, and 851 of them concluded the 5-year follow-up period. Blood pressure control was assessed by office measurements in 439 patients, and by ABPM in 412 patients. In the ABPM group, 20 primary events (total morbidity and mortality and cerebrovascular events) were registered, whereas in the other group, 35 primary events have occurred (p = 0.037). In addition, 22% of patients had white coat hypertension and were excluded from the antihypertensive drug treatment.

Also, Cle ment et al^68^ showed that ambulatory systolic pressure higher than 135 mmHg had a strong correlation with the prognosis of patients treated with antihypertensive therapy, independently of blood pressure measured at the physician's office.

 In relation to the role of ABPM in the guidance of the antihypertensive treatment, further studies to confirm and extend the information that the use of this method will lead to lower morbidity and mortality from arterial hypertension are needed.

One practical issue that remains unsolved is: despite the above considerations about the method to assess blood pressure for 24 hours, how can ABPM be reasonably applied in the clinical practice?

**Figure 5:**
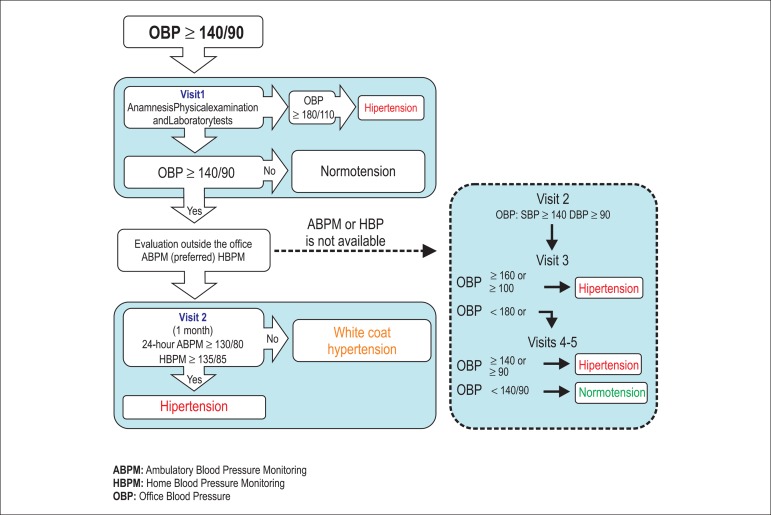
Algorithm suggesting the rational application of ambulatory blood pressure monitoring to evaluate blood pressure behaviors. OBP: office blood pressure; ABPM: ambulatory blood pressure monitoring; HBPM: home blood pressure monitoring; SBP: systolic blood pressure/ DBP: diastolic blood pressure.

To answer this question, we suggest a number of evaluations, based on the algorithm of the Canadian guidelines^69^ for the use of ABPM to identify blood pressure behaviors (Figure 5).

### Perspectives

Similarly to casual blood pressure measures, which started to be used in the end of 19th century when technique and criteria of normality were unknown, and above all, the benefits of measuring blood pressure were not clear, ABPM started to be used in comparable conditions in the end of 20th century and 21st century.

If considerable effort had not been dedicated for the improvement of the method to obtain blood pressure measures using a sphygmomanometer, if reference values had not been obtained by epidemiological studies and their application well established, we would not know even the most basic and essential concepts of the risks of hypertension and the benefits of its control. And this is how we should procedure with ABPM also. A cautious use of the method, based on scientific data supporting the increase in the use of ABPM, will provide the necessary evidence for the extensive use of the method. As a result, the benefits of the method in favor of the understanding of hypertension and necessary care for its treatment will be fully explored.

The analysis of new parameters (other than those classically used today), such as the area under the pressure curve, possibility of evolution of the devices, and use of the ABPM in specific populations should be incorporated to the clinical practice soon.

Cheaper, more reliable and more comfortable monitors, in addition to studies showing the reduction in cardiovascular morbidity and mortality by the ABPM, used in the diagnosis and antihypertensive treatment, should be the near future of 24-hour ABPM.

Therefore, after these considerations, it can be stated that the ABPM is definitely indicated for suspected white coat hypertension, white coat normotension or masked hypertension, and for establishing blood pressure behavior as in hypertension during sleep. In addition, it is the best prognostic marker in different types of blood pressure behavior, with relevant role in the assessment of the antihypertensive treatment.

The studies on blood pressure behavior and its variations during people's daily activity have undoubtedly become less obscure, enlightened by the advent of ABPM, which completes five decades of clinical application and progression.

Therefore, it is fair to say, in light of these data, that the title of this review: "Ambulatory blood pressure monitoring: five decades of more enlightenment and less darkness" is clearly justified.

We believe that, in consonance with the title of this review, the ABPM has shed light to the understanding of blood pressure behaviors in the last five decades, drastically reducing the darkness of the diagnosis of hypertension and blood pressure variations. The ABPM allowed the establishment of the prognosis of patients with altered blood pressure and the assessment of antihypertensive drug treatment in use.
